# Identification of key pseudogenes in nasopharyngeal carcinoma based on RNA-Seq analysis

**DOI:** 10.1186/s12885-021-08211-x

**Published:** 2021-04-30

**Authors:** Xiujuan Zhang, Xiaole Song, Yuting Lai, Bijun Zhu, Jiqin Luo, Hongmeng Yu, Yiqun Yu

**Affiliations:** 1 Department of Otolaryngology, Eye, Ear, Nose and Throat Hospital, Shanghai Key Clinical Disciplines of Otorhinolaryngology, Fudan University, 83 Fen Yang Road, Shanghai, 200031 China; 2 Research Units of New Technologies of Endoscopic Surgery in Skull Base Tumor, Chinese Academy of Medical Sciences, Beijing, 100730 China

**Keywords:** Nasopharyngeal carcinoma, Pseudogene, RNA-Seq, Cytochrome P450, Fc gamma receptor

## Abstract

**Background:**

Nasopharyngeal carcinoma (NPC) is a malignant head and neck tumor, and more than 70% of new cases are in East and Southeast Asia. However, association between NPC and pseudogenes playing important roles in genesis of multiple tumor types is still not clear and needs to be investigated.

**Methods:**

Using RNA-Sequencing (RNA-seq) technology, we analyzed pseudogene expression in 13 primary NPC and 6 recurrent NPC samples as well as their paracancerous counterparts. Quantitative PCR was used to validate the differentially expressed pseudogenes.

**Results:**

We found 251 differentially expressed pseudogenes including 73 up-regulated and 178 down-regulated ones between primary NPC and paracancerous tissues. Enrichment analysis of gene ontology (GO) and Kyoto Encyclopedia of Genes and Genomes (KEGG) pathway were conducted to filter out the key pseudogenes. We reported that pseudogenes from cytochrome P450 (CYP) family, such as *CYP2F2P*, *CYP2G1P*, *CYP4F24P*, *CYP2B7P* and *CYP2G2P* were significantly down-regulated in NPC compared to paracancerous tissues, while *IGHV1OR15–2*, *IGHV3–11*, *FCGR1CP* and *IGHV3–69-1* belonging to Fc gamma receptors were significantly up-regulated. *CYP2B7P*, *CYP2F2P* and *CYP4F26P* were enriched in arachidonic acid metabolism pathway. The qRT-PCR analysis validated the lower expression of pseudogenes *CYP2F2P* and *CYP2B7P* in NPC tissues and cell lines compared to paracancerous tissues and normal human nasopharyngeal epithelial cell line. *CYP2B7P* overexpression weakened migratory and invasive capacity of NPC cell line. Moreover, the expression pattern of those pseudogenes in recurrent NPC tissues was different from the primary NPC.

**Conclusion:**

This study suggested the role of pseudogenes in tumorigenesis and progression, potentially functioning as therapeutic targets to NPC.

**Supplementary Information:**

The online version contains supplementary material available at 10.1186/s12885-021-08211-x.

## Introduction

Nasopharyngeal carcinoma (NPC) is a malignant head and neck tumor arising from the epithelial lining of nasopharynx [1], which possesses the highest incidence rate among all head and neck squamous cell carcinoma (HNSCC) [2]. It has unique ethnic and geographic distribution, whereby more than 70% of new cases are in east and southeast Asia, with an estimated incidence rate in China 7.5 times higher than Europe and North America in 2018 [3]. NPC incidence is higher in males than females in China, with a ratio of about 2.5 in 2015 [4]. Familial aggregation is shown in endemic areas of China and familial NPC patients account for 10% ~ 13% of of the total NPC population in these areas [5, 6]. Three critical factors including genetic susceptibility, Epstein-Barr virus (EBV) infection and chemical carcinogens contributing to the pathogenesis of NPC are illuminated during past decades [7–10]. Multiple significant genes, such as *BCL-2*, *P53*, *LMP-1*, *EGFR* and *PECAM-1* associated with the progression of NPC are identified [11–14]. Although NPC is sensitive to radiotherapy, 10–20% of patients suffer from local recurrence after treatment [15]. Tumor-suppressive microRNA was reported to participate in nearly every step of HNSCC recurrence and metastasis [16]. Long noncoding RNA is differentially expressed between primary and recurrent NPC, exerting impact on NPC recurrence [17].

Traditionally, pseudogenes are considered as non-functional segments of DNA. However, development of high-throughput sequencing technology facilitates the discovery that these gene fossils can regulate gene expression at different levels [18–21]. Moreover, these pseudogenes play important roles in tumorigenesis [22–24]. For instance, *PTENP1* regulates its parent gene *PTEN*, a tumor suppressor in human carcinoma, to inhibit the growth of tumor cells [25] and plays an important role in the initiation and progression of HNSCC [26, 27]. *CYP4Z2P* promotes tumor angiogenesis in breast cancer [28]. *FTH1P3* enhances the proliferation, migration and invasion of oral squamous cell carcinoma and laryngeal squamous cell carcinoma [29, 30]. Zinc ribbon domain containing 1 antisense 1 (*ZNRD1-AS1*) accelerates invasion and metastasis of NPC cells [31]. Besides, pseudogenes contribute to HNSCC progression in vivo [32]. However, there has been few reports describing expression of pseudogenes in NPC yet.

Through RNA-Seq analysis, we proposed that differentially expressed CYP450 and Fc gamma receptor pseudogenes between primary NPC tissues and their paracancerous counterparts might exert impact on tumorigenesis of NPC. These CYP450 pseudogenes participated in arachidonic acid metabolism pathway. Differential CYP450-mRNA levels were also validated between NPC cell lines, including HNE1, CNE1, CNE2, and NP69 normal nasopharyngeal cell line. Overexpression of *CYP2B7P* led to reduction in migratory and invasive HNE1 cells, showing the function of *CYP2B7P* in regulating the NPC malignancy. Expression pattern of above-mentioned pseudogenes were different in recurrent NPC tissue compared to primary NPC. Thus, the differentially expressed pseudogenes in NPC suggested their unique roles in tumorigenesis and progression.

## Materials and methods

### Patient samples

Tumor and paracancerous samples were obtained from 13 patients with primary NPC and 6 patients with recurrent NPC from Eye, Ear, Nose and Throat Hospital, Fudan University. Each tumor sample was paired with paracancerous tissue as control. Primary NPC samples were acquired by endoscopic biopsy, while recurrent NPC samples were acquired from dissected tumors by surgical intervention. All patients with primary NPC were initially diagnosed, and none of them received radiotherapy or chemotherapy before. All patients with recurrent NPC were treated with radiotherapy first and subjected to surgical intervention. Written informed consents were obtained from all patients in accordance with the institutional guidelines and this study was approved by the Institutional Research Ethics Committee of Eye, Ear, Nose and Throat Hospital, Fudan University (Permit Number: 2019081).

### Biopsy and EBV detection

Serum samples of NPC patients were collected to test for anti-EBV IgA antibodies based on patient consent. Serum EBV results were obtained from Clinical Lab of Eye, Ear, Nose and Throat Hospital, Fudan University. Tumor samples were fixed by 10% formalin solution overnight and then embedded in paraffin. Sections at 4 μm thickness were prepared and immunostained with antibodies against CKpan, P63, P40, KI67, LCA, EGFR, P16 and 34βE12, as well as by in situ hybridization for EBV-encoded RNAs (EBERs) to determine the EBV positivity. These data were obtained from Pathology Department of Eye, Ear, Nose and Throat Hospital, Fudan University.

### RNA isolation and RNA-sequencing

Total RNA of each sample was extracted by TRIzol Reagent (ThermoFisher, #15596018) according to the product manual. RNA concentration was measured with NanoDrop2000. RNA-Sequencing (RNA-Seq) analysis was conducted by Majorbio Co. (Shanghai, China). High-quality RNA samples (OD260/280 = 1.8 ~ 2.2, OD260/230 ≥ 2.0, RIN ≥ 6.5, 28S:18S ≥ 1.0, > 2 μg) were used to construct sequencing library. Sequencing reads were mapped to the human reference genome (GRCh38) using HISAT2. The cutoff for differentially expressed genes was set at |log_2_FC| > 2.0. We performed Gene Ontology (GO) and Kyoto Encyclopedia of Genes and Genomes (KEGG) pathway analysis on I-Sanger (www.i-sanger.com). The RNA-Seq data was deposited in NCBI (Accession code GSE134886).

### Cell line culture

Human NPC cell lines HNE1 and CNE1 were obtained from the Sun Yat-sen University Cancer Center (Guangzhou, China). The cell lines were authenticated by PCR analysis targeting the E6/E7 sequences of HPV18 and tested for mycoplasma contamination before further analysis [33]. Normal nasopharyngeal epithelial cell line NP69 was obtained from the Chinese Academy of Science (Beijing, China). As previously described [34], Cells were cultured in RPMI-1640 medium with 10% fetal bovine serum (FBS) and 100 U/ml penicillin–streptomycin (ThermoFisher), and grown in a humidified atmosphere of 5% CO_2_ and 95% air.

### Quantitative real time PCR

Total RNA was extracted by TRIzol Reagent. RNA was reversely transcribed to cDNA using PrimeScript RT reagent Kit with gDNA Eraser (Takara, RRO47A). Primers were synthesized by JIE LI BIOLOGY Co. (Shanghai, China). Quantitative real time PCR was performed on Applied Biosystems 7500 using SYBR Green qPCR SuperMix (E096-01B, Novoprotein). *GAPDH* was used as the normalization control. The relative expression levels were calculated using 2^-△△Ct^ method. Primer sequences were as follows: *GAPDH*: forward GGAGCGAGATCCCTCCAAAAT, reverse GGCTGTTGTCATACTTCTCATGG. *CYP4F26P*: forward TGTAGGTGGTTGCCGTTTGT, reverse CAGGGCCATCTGTGGATGTT. *CYP2F2P*: forward, TGGGAGTGGTCATTGTCTACC, reverse AGCACTTGACGCACAGTAGG. *IGHV3–69-1*: forward GGTCCCTGAGACTCTCCTGT, reverse GTGAATCGGCCCTTCACAGA. *FCGR1CP*: forward CACTACACATCAGCAGGAATA TCAC, reverse TCACAGCTCAGGGTGACCA.

### Cell transfection

The full length of *CYP2B7P* was cloned and inserted in plasmid pEGFP-N2. HNE1 cells at 80–90% confluence were transfected with *CYP2B7P* construct and empty pEGFP-N2 plasmid using Lipofectamine 2000 (ThermoFisher). Approximately 48 h after transfection, cells were collected by fluorescence-activated cell sorting (MoFlo XDP Cell Sorter, Beckman Coulter) based on green fluorescent protein (GFP). Sorted cells were subjected to further analysis.

### Migration and invasion assays

For migration assay, sorted cells were suspended with serum-free RPMI-1640 medium, making a final density of 1–2х10^5^ cells/mL. 100 μL of cell suspension was seeded in the upper chamber of a transwell plate (Corning #3422) and 600 μL RPM1–1640 medium with 10% FBS was added in the lower chamber. After 24-h incubation, cells were fixed with 4% paraformaldehyde for 20 min and stained with crystal violet for 3 min. The cells in the upper chamber were removed with a cotton swab. The migrated cells on the lower surface were photographed with Leica DMi8 and counted with Image J software. For invasion assay, all the procedures were as the same as migration assay except that upper chamber was precoated with 100 μL of Matrigel (Corning #356231).

### Statistical analysis

All experiments were performed in triplicate. Paired or unpaired t test was used to analyze the differences between two groups using Graphpad Prism software. *P* value < 0.05 was considered as statistically significant.

## Results

### Clinicopathologic features of NPC patients

The clinical characteristics of 13 patients with primary NPC and 6 patients with recurrent NPC from whom tumors and paracancerous tissues were collected were summarized in Table 1. Primary NPC samples were collected from eight male and five female patients, with an average age of 51 years (ranging from 33 to 75 years old), while samples of recurrent NPC were collected from 6 males, with an average of 54 years (ranging from 47 to 61 years old). Approximate 77% of the patients with primary NPC (10/13) were diagnosed with non-keratinizing carcinoma and undifferentiated carcinoma (type II and type III NPC), while 23% (3/13) were diagnosed with squamous cell carcinoma (type I NPC). 67% (4/6) of the recurrent NPC patients were diagnosed with squamous cell carcinoma. All primary NPC samples (13/13) were positively immunostained with CKpan, P63, P40, KI67 and EGFR, but negatively with LCA. In situ hybridization for EBER showed that all samples were positive except for one recurrent NPC patient. All the serum samples were EBV IgA^+^.

**Table 1:** Clinical features of patients with NPC

	Patient	Age	Gender	Diagnosis	CKpan	P63	P40	KI67	LCA	EGFR	P16	34βE12	EBER	EBV
**Primary NPC**	SXL	49	F	typeII,typeIII	+	+	*	30–40%+	–	+	–	*	+	*
	HCH	65	M	typeII,typeIII	+	+	*	50%+	–	+	–	+	+	+
	WCZ	33	M	typeII,typeIII	+	+	+	60–70%+	–	+	–	+	+	*
	JYT	57	M	typeII,typeIII	+	+	partial +	10–20%+	–	+	–	+	+	+
	LSB	75	M	typeII,typeIII	+	+	+	40%+	–	+	–	+	+	+
	ZLZ	61	F	typeI	+	+	+	30–40%+	–	+	partial +	+	+	+
	CMW	70	M	typeI	+	+	+	40%+	–	+	+	+	+	*
	ZQX	70	F	typeII,typeIII	+	+	+	70%+	–	+	–	+	+	*
	WHW	40	M	typeII,typeIII	+	+	+	20%+	–	+	partial +	+	+	+
	XZP	37	F	typeII,typeIII	+	+	+	30%+	–	+	–	+	+	+
	GSW	51	M	typeII,typeIII	+	partial +	partial +	40%+	–	+	–	+	+	+
	ZXE	51	F	typeI	+	+	partial +	30%+	–	+	–	+	+	+
	LYG	42	M	typeII,typeIII	+	+	partial +	60%+	–	+	–	+	+	+
**Recurrent NPC**	LJQ	55	M	typeII,typeIII	+	+	+	40%+	*	+	–	*	+	*
	ZXF	61	M	typeI	+	+	+	50%+	–	+	*	*	+	*
	LWM	56	M	typeII,typeIII	+	+	+	70–80%+	*	+	–	+	+	*
	WJL	49	M	typeI	+	+	+	50%+	*	*	*	*	+	*
	ZCZ	47	M	typeI	+	+	+	50%+	*	*	–	*	–	+
	PJ	54	M	typeI	+	+	+	70%+	*	+	*	*	+	*

TypeI: squamous cell carcinoma, type II: non-keratinizing carcinoma, type III: undifferentiated carcinoma. + represented positive result while - represented negative result. * meant lacking related data. *LCA* Leukocyte common antigen, *EGFR* Epidermal growth factor receptor, *EBV* Epstein-Barr virus, *EBER* EBV-encoded RNA

### Evaluation of NPC and paracancerous tissue transcriptome sequencing

The clean reads of each sample were separately aligned to reference genome. We obtained more than 6.14 Gb clean data from each sample, and the Q30 base of each sample was over 92.76%. Transcripts per kilobase million (TPM) was used to normalize the transcriptions (Fig.S1a). The proportion of unique reads that mapped to GRCh38 ranged from 83.67 to 95.86%. A high consistency of each sample was shown by the correlation of matrix (R^2^>0.9, Fig.S1b). Principal component analysis (PCA) was carried out to estimate the clustering nature of these samples, showing that NPC and paracancerous samples were differentially clustered (Fig.S1c). A total of 41,715 genes were detected, from which 4358 genes were differentially expressed between NPC and paracancerous tissues, including 1766 upregulated and 2592 downregulated genes (Fig.S1d).

### Annotation of differentially expressed pseudogenes

**Fig. 1:**
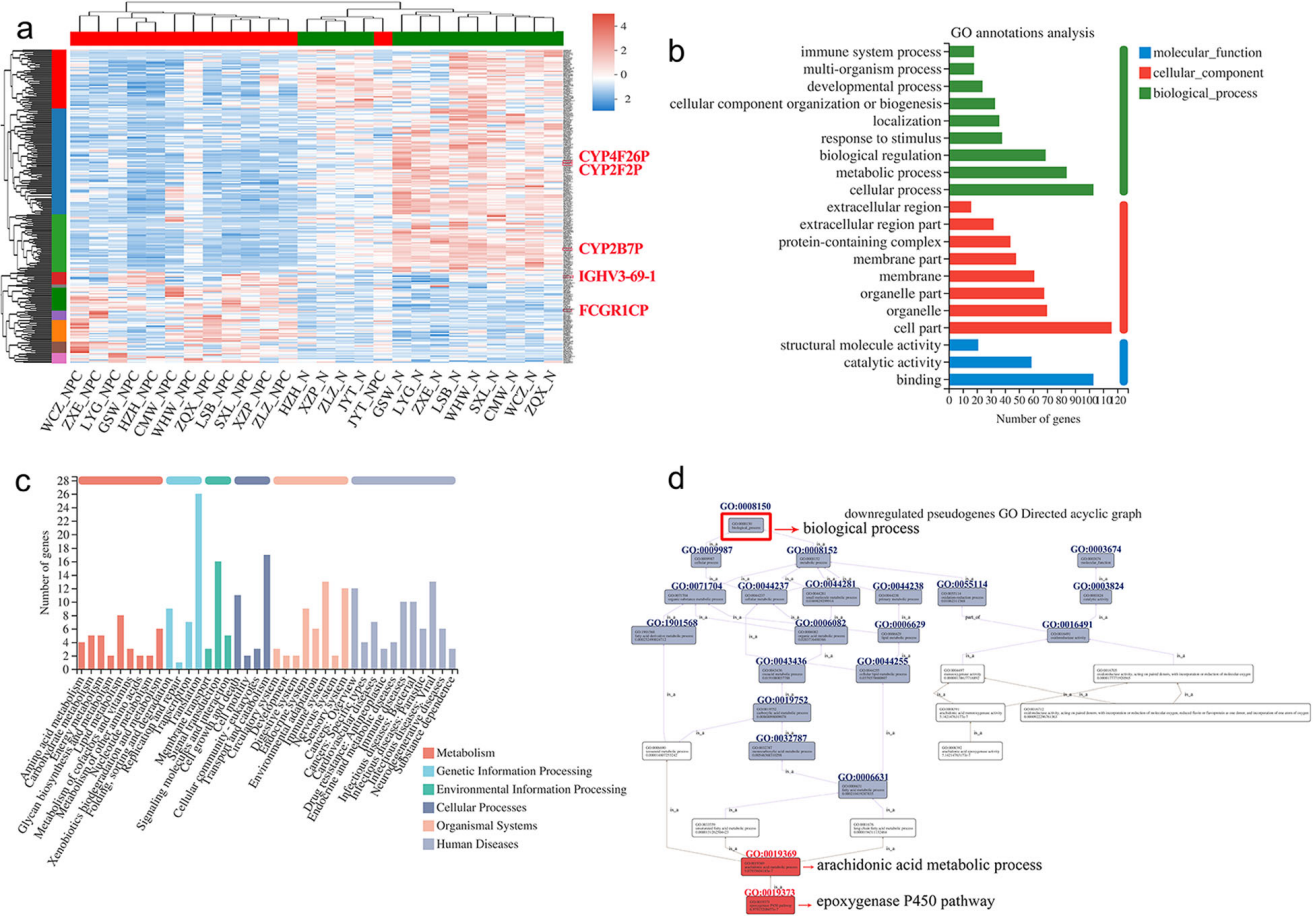
Annotation of differentially expressed pseudogenes between NPC and paracancerous tissues. **a** Heatmap of 251 differentially expressed pseudogenes (73 up-regulated and 178 down-regulated genes). N represented paracancerous control. Genes highlighted in red were subjected to further analysis. The gene information was listed in Table S1 and S2. **b**, **c** GO and KEGG annotations of differentially expressed pseudogenes. **d** Analysis of GO directed acyclic graph on the downregulated pseudogenes. The key GO items were highlighted by red in (**d**). KEGG is developed by Kanehisa Laboratories [35]

Among 4358 differentially expressed genes (DEGs), 251 pseudogenes (5.8%) were identified, including 73 up-regulated and 178 down-regulated pseudogenes. Clustering analysis result was shown in Fig. 1a and complete gene information was listed in Table S1 and S2. We applied GO and KEGG annotations to classify these differentially expressed pseudogenes into different terms and pathways. GO terms were classified into three ontologies, including biological process (BP), cellular component (CC) and molecular function (MF) (Fig. 1b). Immune system process (GO:0002376), multi-organism process (GO:0051704), developmental process (GO:0032502) and other top 20 quantification of GO annotation terms were shown in Fig. 1b. KEGG functional annotations were classified into six subtypes, including metabolism (M), genetic information processing (GIP), environmental information processing (EIP), cellular processes (CP), organismal systems (OS) and human diseases (HD) (Fig. 1c). KEGG pathways included amino acid metabolism, immune system and immune disease pathways, etc. (Fig. 1c). In addition, the downregulated pseudogenes were analyzed with GO directed acyclic graph, indicating that epoxygenase P450 pathway was a subtype of arachidonic acid metabolic process belonging to BP (Fig. 1d).

### Function classification of differentially expressed pseudogenes

**Fig. 2:**
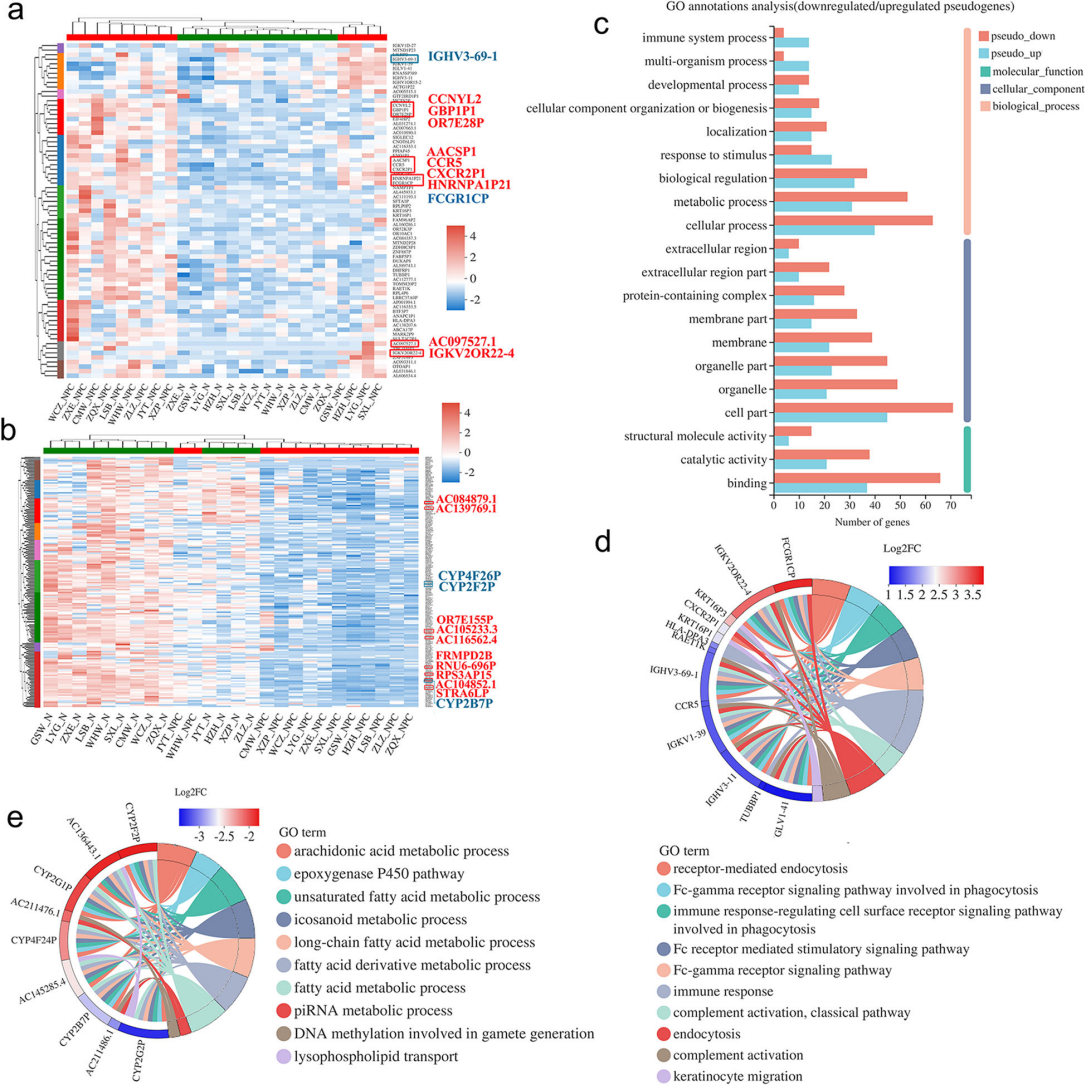
GO analysis of up- and down-regulated pseudogenes. **a**, **b** Heatmaps of up- and down-regulated pseudogenes. Top 20 differentially expressed genes were highlighted and genes in cyan were further analyzed. Information of the top 20 differentially expressed genes was listed in Table S3 and S4. **c** GO annotation showed the top 20 terms. **d**, **e** GO chord plot analysis showed the significantly up- and down-regulated pseudogenes with their involved GO terms

We then compared the up- and down-regulated pseudogenes to investigate their biological functions in detail. DEGs with similar expression levels were clustered together and marked with the same color. The top 10 upregulated pseudogenes were *HNRNPA1P21*, *AACSP1*, *FCGR1CP*, *CCR5*, *CXCR2P1*, *AC097527.1*, *OR7E28P*, *CCNYL2*, *IGKV2OR22–4* and *GBP1P1* (Fig. 2a, marked by rectangles), while the top 10 downregulated ones were *STRA6LP*, *AC084879.1*, *AC116562.4*, *RNU6-696P*, *OR7E155P*, *RPS3AP15*, *AC104852.1*, *FRMPD2B*, *AC139769.1* and *AC105233.3* (Fig. 2b, marked by rectangles). Details of the 10 most significantly up- and down-regulated pseudogenes were listed in Table S3 and S4.

We then performed GO analysis on up- and down-regulated pseudogenes, based on GO annotation terms. Both up- and down-regulated pseudogenes were classified into GO terms of immune system process (GO:0002376), multi-organism process (GO:0051704), developmental process (GO:0032502), cellular component organization or biogenesis (GO:0071840), localization (GO:0051179), response to stimulus (GO:0050896) and other GO annotations (Fig. 2c). The top 20 enriched GO terms of up-regulated pseudogenes belonged to BP and MF classes, while 18 of the top 20 GO terms were associated with BP class of Fc-gamma receptor, immune system process and other related signaling pathway terms (Fig.S2). The significantly up-regulated pseudogenes including *FCGR1CP*, *IGKV2OR22–4*, *IGHV3–69-1*, *IGKV1–39*, *IGHV3–11* and *IGLV1–41* were highly enriched into 7 GO terms, including receptor-mediated endocytosis (GO:0006898), Fc-gamma receptor signaling pathway involved in phagocytosis (GO:0038096), immune response-regulating cell surface receptor signaling pathway involved in phagocytosis (GO:0002433), Fc receptor mediated stimulatory signaling pathway (GO:0002431), Fc-gamma receptor signaling pathway (GO:0038094), immune response (GO:0006955) and endocytosis (GO:0006897) (Fig. 2d). The significantly down-regulated pseudogenes *CYP2F2P*, *CYP2G1P*, *CYP4F24P*, *CYP2B7P* and *CYP2G2P* were enriched into another 7 GO terms, including arachidonic acid metabolic process (GO:0019369), epoxygenase P450 pathway (GO:0019373), unsaturated fatty acid metabolic process (GO:0033559) and others that were shown in GO chord plot (Fig. 2e). Epoxygenase P450 pathway was a subtype of arachidonic acid metabolic process of BP (Fig. 1d). Therefore, these data suggested the important role of epoxygenase P450 pathway in the biological progression of NPC.

### KEGG pathway enrichment analysis of differentially expressed pseudogenes

**Fig. 3:**
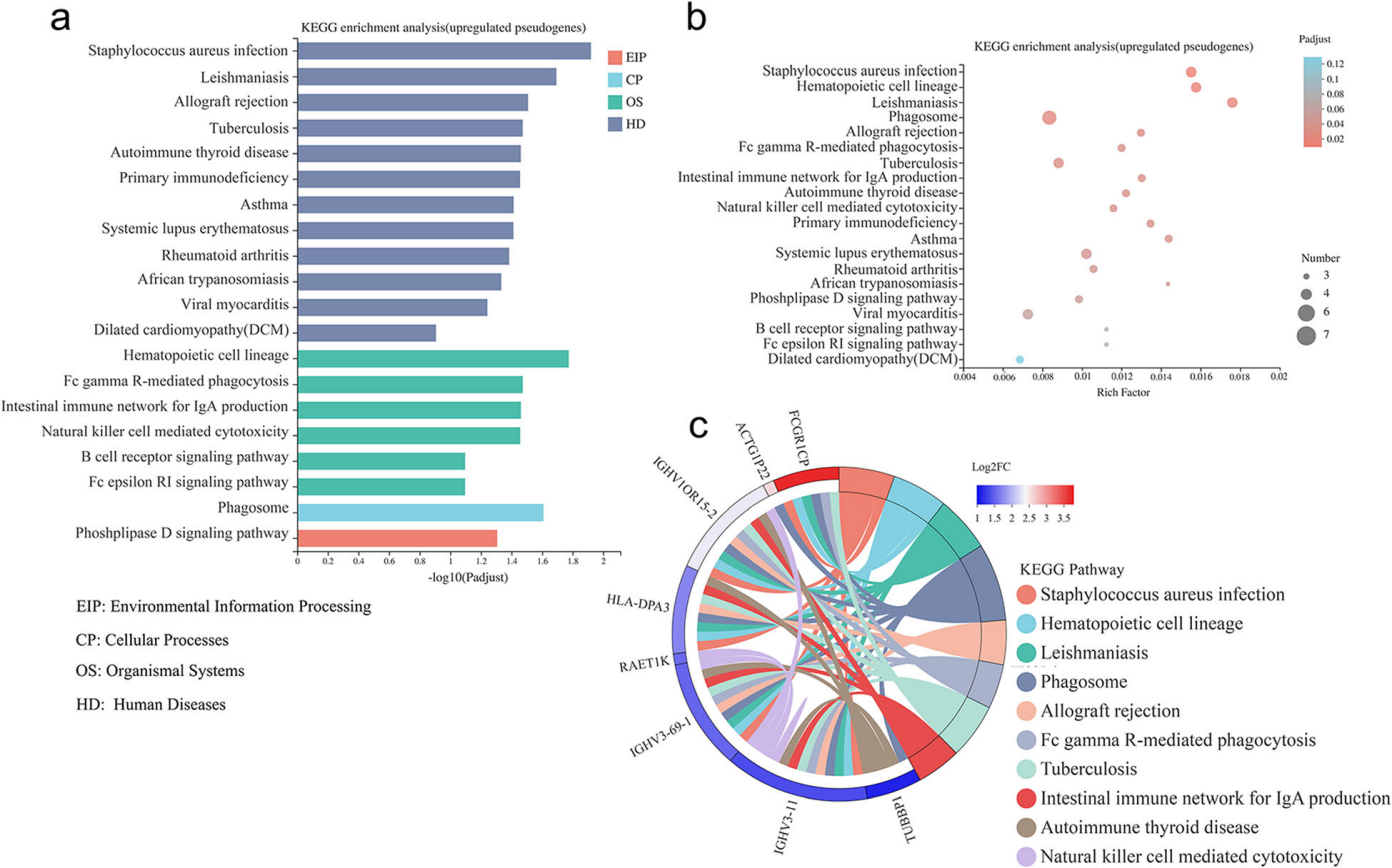
KEGG enrichment analysis of up-regulated pseudogenes. **a** The top 20 KEGG enrichment pathways. **b** Functional categorization of top 20 KEGG enrichment pathways. **c** KEGG chord plot analysis showed the significantly up-regulated pseudogenes. KEGG is developed by Kanehisa Laboratories [35]

We performed KEGG pathway enrichment analysis on differentially expressed pseudogenes and filtrated out three most statistically significant pathways participated by down-regulated pseudogenes, including arachidonic acid metabolism (map 00590), olfactory transduction (map 04740) and fat digestion and absorption (map 04975). The down-regulated pseudogenes involved in arachidonic acid metabolism pathway were *AC136433.1*, *CYP4F26P*, *AC145285.4*, *AL390726.5* and *CYP2B7P* (Fig.S3). The top 20 KEGG enrichment pathways of up-regulated pseudogenes included leishmaniasis, phagosome, Fc gamma R-mediated phagocytosis, natural killer cell mediated cytotoxicity and B cell receptor signaling pathways (Fig. 3a). The rich factor of these 20 pathways were analyzed and Leishmaniasis pathway was the most significant enriched pathway with a rich factor of 0.018, while Phagosome pathway enriched the maximum number of pseudogenes (Fig. 3b). The significantly up-regulated pseudogenes in KEGG chord plot included *HLA-DPA3*, *IGHV1OR15–2*, *IGHV3–11*, *FCGR1CP* and *IGHV3–69-1* (Fig. 3c), while *FCGR1CP* and *IGHV3–69-1* were significantly up-regulated in GO chord plot as well (Fig. 2d).

### Differentially expressed pseudogenes between NPC and paracancerous counterparts

Results from GO term enrichment analysis on differentially expressed pseudogenes were consistent with KEGG pathway enrichment analysis in both up- and down-regulated pseudogenes. These data showed that immunoglobulin heavy variable-region (IGHVR) related pseudogenes were up- regulated, while cytochrome P450 family related pseudogenes were down-regulated in NPC compared to paracancerous tissues (Figs. 2 and 3). Significantly up-regulated pseudogenes such as *IGHV1OR15–2*, *IGHV3–11*, *FCGR1CP* and *IGHV3–69-1* belonged to or regulated Fc gamma receptor. However, it failed to form a correlation network when we plotted the correlation among upregulated *HLA-DPA3*, *IGHV1OR15–2*, *IGHV3–11*, *FCGR1CP* and *IGHV3–69-1* genes, suggesting lack of association among these pseudogenes.

**Fig. 4:**
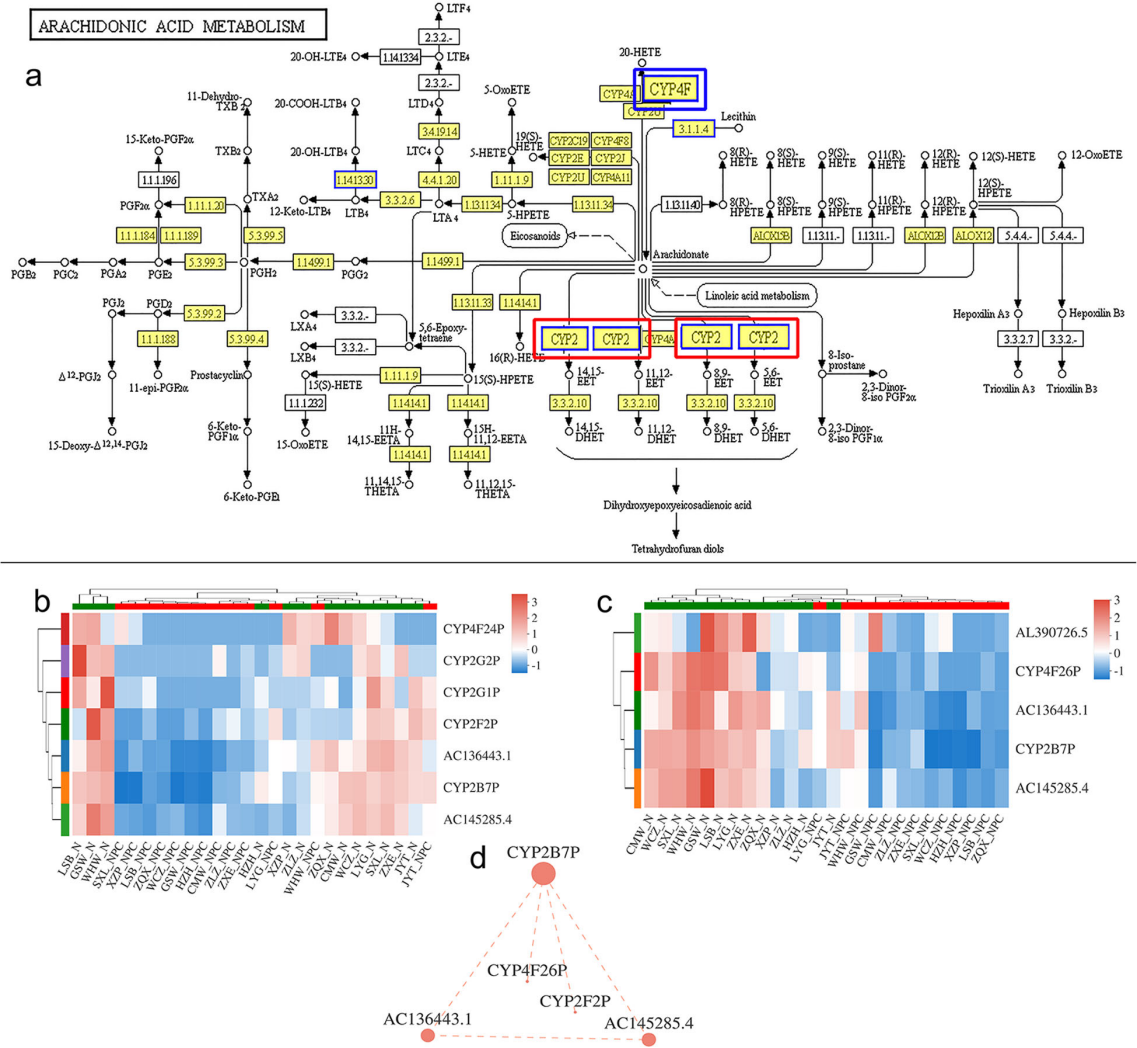
CYP450 pseudogenes participated in arachidonic acid metabolism. **a** A scheme showing arachidonic acid metabolism pathway. Red boxes indicated the location of *CYP2F2P* and *CYP2B7P*, and blue box showed the location of *CYP4F46P*. **b** Heatmap of CYP450 pseudogenes involving in arachidonic acid metabolic process by GO enrichment analysis. **c** Heatmap of pseudogenes involving in arachidonic acid metabolism pathway by KEGG enrichment analysis. **d** The correlation among *CYP2B7P*, *CYP2F2P*, *CYP4F26P*, *AC136443.1* and *AC145285.4*. KEGG is developed by Kanehisa Laboratories [35]

Significantly down-regulated pseudogenes, such as *AC136433.1*, *CYP4F26P*, *AC145285.4*, *AL390726.5* and *CYP2B7P* were involved in epoxygenase P450 pathway and participated in arachidonic acid metabolic process (Figs. 1d and 4a). These down-regulated pseudogenes were mapped into the arachidonic acid metabolism KEGG pathway (Fig. 4a), implying their functional locations in the whole signaling network. Moreover, the expression patterns of *CYP2B7P* and *CYP2F2P* were similar in GO term of arachidonic acid metabolic process (Fig. 4b), consistent with the KEGG result and the expression level of *CYP2B7P* was similar as *CYP4F26P* in KEGG enrichment result of arachidonic acid metabolism pathway (Fig. 4c). In order to validate this hypothesis, we plotted correlation among *CYP2B7P* and *CYP4F26P*, *CYP2F2P*, *AC136443.1* and *AC145285.4*, illustrating a constitutive association among expression levels of *CYP2B7P*, *CYP2F2P*, *CYP4F26P*, *AC136443.1* and *AC145285.4* (Fig. 4d, R^2^>0.8). Thus, *CYP2F2P, CYP2B7P* and *CYP4F26P* were key pseudogenes participating in the arachidonic acid metabolism pathway, potentially regulating the tumorigenesis of NPC.

**Fig. 5:**
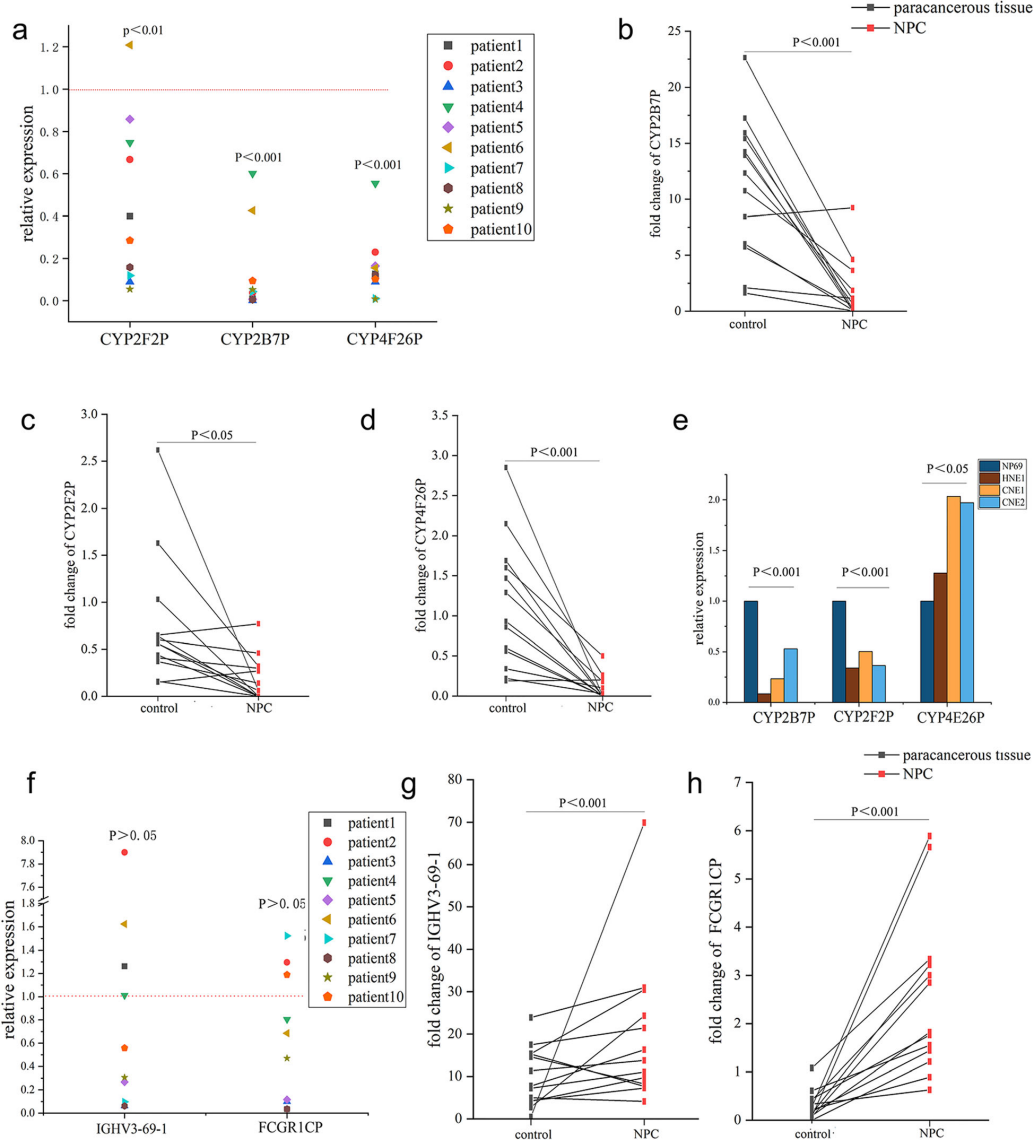
Key pseudogenes differentially expressed in primary NPC tissues and cell lines compared to controls. **a** Quantitative PCR data showed the reduction of *CYP2F2P*-, *CYP2B7P*- and *CYP4F26P*-mRNA levels in NPC compared to paracancerous tissues. **b**-**d** Fold change of *CYP2B7P*, *CYP2F2P* and *CYP4F26P* by RNA-Seq analysis. **e** Quantitative PCR analysis on *CYP2B7P*-, *CYP2F2P*- and *CYP4F26P*-mRNA levels in NPC and normal nasopharyngeal epithelial cell lines. **f** *IGHV3–69-1* and *FCGR1CP* expression levels in NPC and paracancerous tissues. **g**, **h** Fold change of *IGHV3–69-1* and *FCGR1CP* by RNA-Seq analysis. The statistical difference was determined by paired t test

**Fig. 6:**
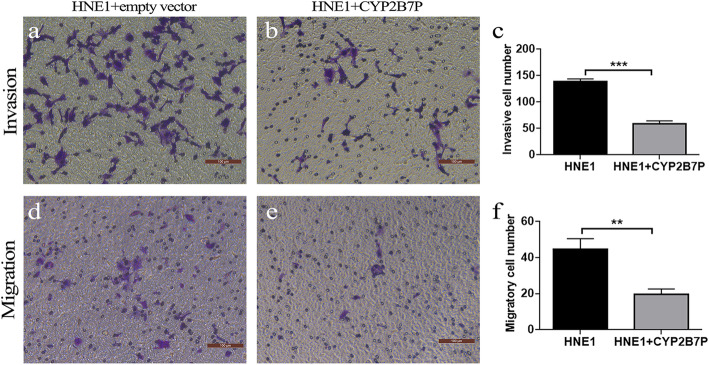
CYP2B7P overexpression inhibited migration and migration of HNE1 cells. **a**, **b** Images of invasive HNE1 cells transfected with empty vector and CYP2B7P construct. **c** Statistical analysis of invasive cells. **d**, **e** Images of migratory HNE1 cells. **f** Statistical analysis of migratory HNE1 cells transfected with empty vector and CYP2B7P construct. Statistical significance was determined by unpaired t test. **p < 0.01, ***p < 0.001. Scale bars: 100 μm

To validate the RNA-Seq data, expression of *CYP2B7P*, *CYP2F2P* and *CYP4F26P* that were involved in arachidonic acid metabolism, as well as *IGHV3–69-1* and *FCGR1CP* participating in Fc gamma related immune system were detected by qRT-PCR in 10 pairs of NPCs and their paracancerous samples, as well as in NPC cell lines (HNE1, CNE1 and CNE2) and NP69 normal human nasopharyngeal epithelial cell line. Levels of *CYP2B7P*-, *CYP2F2P*- and *CYP4F26P*-mRNA were decreased in almost all NPC tumors compared to paracancerous tissues (Fig. 5a), consistent with the data obtained from the RNA-Seq analysis (Fig. 5b-d). However, we only found the lower expression levels of *CYP2B7P* and *CYP2F2P* in NPC cell lines, while no significant reduction in *CYP4F26P*-mRNA level was found in NPC cell lines compared to normal nasopharyngeal epithelial cell line (Fig. 5e). Furthermore, overexpression of *CYP2B7P* decreased the number of migratory and invasive HNE1 cells by 57 ± 3% (*p* < 0.001) and 55 ± 13% (*p* < 0.01) compared to empty vector-transfected control groups (Fig. 6). Levels of up-regulated pseudogenes *IGHV3–69-1* and *FCGR1CP* in NPC tissues were not fully consistent with the RNA-seq data since some NPC tissues showed apparent reduction in *IGHV3–69-1* and *FCGR1CP*-mRNA levels (Fig. 5f-h). Collectively, we displayed key pseudogenes differentially expressed between NPC and paracancerous tissues and *CYP2B7P* expression might regulate malignancy of NPC.

### Primary and recurrent NPC samples showed different patterns in pseudogene expression

**Fig. 7:**
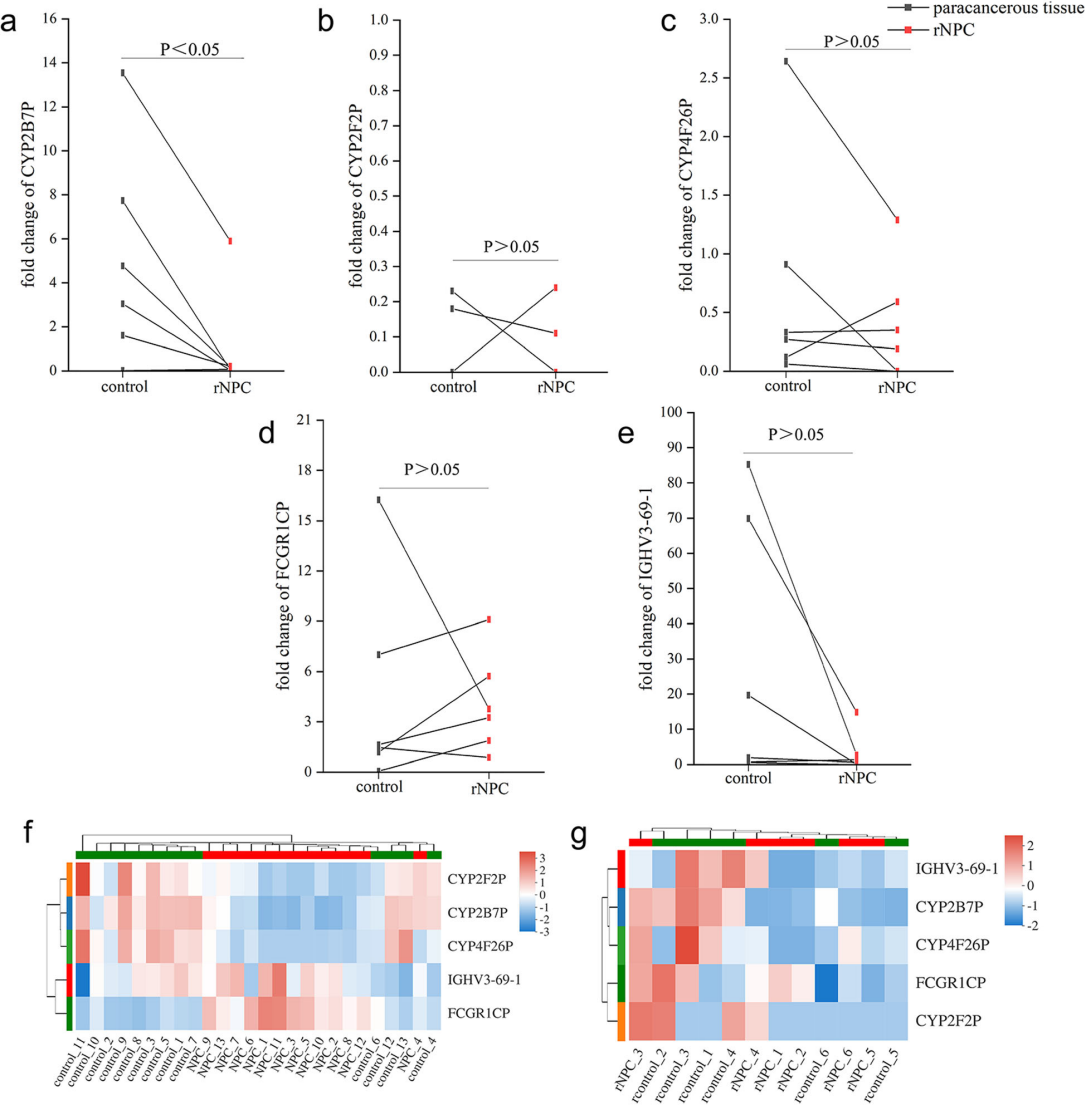
Pseudogene expression pattern in recurrent NPC. **a**-**e** Fold change of *CYP2B7P*, *CYP2F2P*, *CYP4F26P*, *IGHV3–69-1* and *FCGR1CP* in recurrent NPC compared to paracancerous tissues by RNA-seq analysis. **f**, **g** Heatmaps of *CYP2B7P*, *CYP2F2P*, *CYP4F26P*, *IGHV3–69-1* and *FCGR1CP* expression in primary and recurrent NPC samples. The statistical difference was determined by paired t test

Furthermore, we determined whether the expression pattern of above-mentioned pseudogenes was different in recurrent NPC in contrast to primary NPC samples by RNA-Seq analysis. Compared to paracancerous tissues, recurrent NPC samples only showed the significant reduction in fold change of *CYP2B7P*, while fold change of *CYP2F2P*, *CYP4F26P*, *FCGR1CP* and *IGHV3–69-1* was not significantly altered (Fig. 7a-e, g), different from the expression pattern in primary NPC (Figs. 5b-d, g, h and 7f). These data demonstrated that pseudogene expression pattern was different between primary and recurrent NPC tissues, potentially suggesting the differential roles of these pseudogenes in tumorigenesis of primary NPC and progression of recurrent NPC.

## Discussion

In this study, we detected 251 differentially expressed pseudogenes between primary NPC and paracancerous control tissues. CYP450 pseudogenes were significantly down-regulated, while pseudogenes of Fc gamma receptors were up-regulated in NPC tissues. Expression of specific pseudogene regulated the migration and invasion of NPC cells. The expression pattern of these pseudogenes in recurrent NPC was different from primary NPC samples. Thus, pseudogenes might participate in the tumorigenesis of NPC.

The human CYP gene superfamily has 57 functional genes and 58 pseudogenes [36]. Some of them contribute to the activation of pre-carcinogens with genetic polymorphism that have been implicated in susceptibility to NPC [37]. However, reports from different groups are inconsistent, since expression of CYP450 genes in NPC tissues in contrast to non-NPC tissues varies from statistically significant to not significant [37, 38]. To our knowledge, only *CYP2E1* gene contributes to the susceptibility of NPC [39–42]. The role of CYP450 pseudogenes in other tumor types is relatively clear. For instance, *CYP4Z2P* maintains the stemness of breast cancer cells [43, 44], and pseudogene *CYP2A7* affects *CYP2A6* expression in human liver [45]. However, very limited study reported whether CYP450 pseudogenes were associated with NPC tumorigenesis. Here, we found that CYP450 pseudogenes including *CYP2F2P*, *CYP4F26P* and *CYP2B7P* were significantly down-regulated in NPC compared to paracancerous tissues by RNA-Seq analysis (Fig. 2). These data were further confirmed by quantitative PCR in patient samples and NPC cell lines (Fig. 5). *CYP2F2P* is associated with the risk of chronic obstructive pulmonary disease [46]. Lower expression of *CYP2B7P* is associated with prognosis of lung adenocarcinoma [47]. *CYP4F26P* is an independent prognostic indicator for lung squamous cell carcinoma [48]. Combined with our data that *CYP2B7P* functioned in the migration and invasion of NPC cells (Fig. 6), these findings suggest that CYP450 pseudogenes are correlated with disease diagnosis and prognosis. Our work provided evidence to support the potential role of CYP450 pseudogenes in NPC tumorigenesis, thus supplementing the function of CYP450 in cancer pathogenesis and progression. Based on the single cell RNA-Seq data provided by Chen et al. [49], *CYP4F26P* expresses in the epithelial and T cells on NPC tumors (Fig.S4, the data were obtained from db.cngb.org/npcatlas). NPC epithelial cells expresses EBV-DNase, a biomarker in NPC biopsies [50]. Regulatory T cells in NPC regulated antitumor immunity [51]. Tumor-specific CD8+ T cell responses in EBV+ NPC is enhanced through therapeutic targeting of regulatory T cells [52]. Thus, epithelial and T cells on NPC tumors are important for the carcinoma malignancy and therapy. The differential expression of *CYP4F26P* between NPC and paracancerous tissues is likely associated with NPC tumorigenesis and progression.

Critical factors taking part in arachidonic acid metabolism regulate tumor growth and therapeutic resistance, such as Acyl-CoA synthetase 4 (*ACSL4*) in prostate tumor [53], hypermethylated genes in esophageal squamous cell carcinoma [54], the coding gene of epoxide hydrolase 2 *(EPHX2*) in prostate cancer [55], 12-lipoxygenase (*12LOX*) in ovarian cancer [56], etc. Thus, arachidonic acid metabolic pathway participates in the development of malignancies. However, the role of arachidonic acid metabolism in NPC pathogenesis has not been well studied yet. The polymorphism of Cyclooxygenase-2 (Cox-2), a key enzyme in the conversion of arachidonic acid to prostaglandins is proved to mediate susceptibility to NPC [57]. Our data showed that down-regulated CYP450 pseudogenes were mapped into the arachidonic acid metabolism pathway, suggesting the potential role of arachidonic acid pathway in regulation of NPC tumorigenesis. However, more direct evidence is required to verify the role of critical pseudogenes participating in arachidonic acid metabolism in the NPC malignancy.

All of the Fc-gamma receptors belong to the immunoglobulin superfamily [58]. The role of Fc gamma receptor in NPC pathogenesis has not been well elucidated, although the function of Fc gamma receptor on the chemotherapeutic effect against head and neck squamous cell carcinoma cells was reported [59]. Here, we found that *FCGR1CP*, Fc fragment of IgG receptor and *IGHV3–69-1*, immunoglobulin heavy variable were up-regulated in NPC samples compared to paracancerous tissues. Currently, these two pseudogenes have not been reported to play a role in NPC tumorigenesis or progression. Due to the increased level of these pseudogenes in NPC tissues, they might serve as novel biomarkers for precision diagnosis for patients with NPC.

Primary and recurrent NPC samples show differential gene expression patterns, such as kallikrein related peptidase 11 (*KLK11*) [60], succinate dehydrogenase subunit B (*SDHB*), pyruvate dehydrogenase kinase 1(*PDK1*) [61], replication protein A3 (*RPA3*) [62], short palate, lung, nasal epithelium clone1 (*SPLUNC1*) and mixed Lineage Leukemia 3 (*MLL3*) [63] as well as long noncoding RNAs [17]. Thus, the differential pseudogenes expression pattern might lead to the NPC relapse. Here, we found the expression pattern of pseudogenes such as *CYP2F2P, CYP4F26P, FCGR1CP* and *IGHV3–69-1* was different between primary and recurrent NPCs (Fig. 7). This potentially suggested the specific roles of these pseudogenes in NPC recurrence, implying for the possible therapeutic targets to NPC.

## Conclusion

In summary, we displayed a RNA-seq analysis on pseudogene expression in NPC and paracancerous tissues. Levels of CYP450 pseudogenes were decreased while Fc gamma receptor expression was increased in NPC samples compared to paracancerous tissues. The pseudogene expression pattern was different in primary NPC samples from recurrent NPC. Our study suggested that pseudogenes might play specific roles in NPC tumorigenesis and progression.

## Supplementary Information

**Additional file 1: Figure S1.** The transcriptome sequencing data of NPC and paracancerous tissues. **Figure S2.** The top 20 enriched GO terms of up-regulated pseudogenes belonged to BP and MF class. **Figure S3.** KEGG pathway enrichment analysis on down-regulated pseudogenes. Figure 4. *CYP4F26P* expressed in the epithelial and T cells on NPC tumors (Data source: db.cngb.org/npcatlas by Chen et al. [46]). **Table S1.** Down-regulated pseudogenes between primary NPC and paracancerous samples. **Table S2.** Up-regulated pseudogenes between primary NPC and paracancerous samples. **Table S3.** The 10 most significantly down-regulated pseudogenes between primary NPC and paracancerous samples. **Table S4.** The 10 most significantly up-regulated pseudogenes between primary NPC and paracancerous samples.

## Data Availability

The datasets generated during and/or analysed during the current study are available from the corresponding author on reasonable request. The RNA-Seq data was deposited in NCBI (Accession code GSE134886).

## Abbreviations

NPC: Nasopharyngeal carcinoma
RNA-seq: RNA-Sequencing
GO: Gene ontology
KEGG: Kyoto Encyclopedia of Genes and Genomes
CYP: Cytochrome P450
EBV: Epstein-Barr virus
EBERs: EBV-encoded RNAs
DEG: Differentially expressed gene
BP: Biological process
CC: Cellular component
MF: Molecular function
IGHVR: Immunoglobulin heavy variable-region
HNSCC: Head and neck squamous cell carcinoma
