# Supplementary material for: Identification of key pseudogenes in nasopharyngeal carcinoma based on RNA-Seq analysis
Source: BMC Cancer. 2021 Apr 30;21:483. doi: 10.1186/s12885-021-08211-x (PMC8088053; doi:10.1186/s12885-021-08211-x)
Supplement: Supplementary file 1 — Additional file 1: Figure S1. The transcriptome sequencing data of NPC and paracancerous tissues. Figure S2. The top 20 enriched GO terms of up-regulated pseudogenes belonged to BP and MF class. Figure S3. KEGG pathway enrichment analysis on down-regulated pseudogenes. Figure 4. CYP4F26P expressed in the epithelial and T cells on NPC tumors (Data source: db.cngb.org/npcatlas by Chen et al. [46]). Table S1. Down-regulated pseudogenes between primary NPC and paracancerous samples. Table S2. Up-regulated pseudogenes between primary NPC and paracancerous samples. Table S3. The 10 most significantly down-regulated pseudogenes between primary NPC and paracancerous samples. Table S4. The 10 most significantly up-regulated pseudogenes between primary NPC and paracancerous samples. [file 12885_2021_8211_MOESM1_ESM.docx]

Supplementary Materials for

Identification of Key Pseudogenes in Nasopharyngeal Carcinoma Based on RNA-Seq Analysis

Xiujuan Zhang^1^, Xiaole Song^1^, Yuting Lai^1^, Bijun Zhu^1^, Jiqin Luo^1^, Hongmeng Yu^1,2,*^, Yiqun Yu^1,*^

1 Department of Otolaryngology, Eye, Ear, Nose and Throat Hospital, Shanghai Key Clinical Disciplines of Otorhinolaryngology, Fudan University, Shanghai, China 200031.

2 Research Units of New Technologies of Endoscopic Surgery in Skull Base Tumor, Chinese Academy of Medical Sciences, Beijing, China 100730.


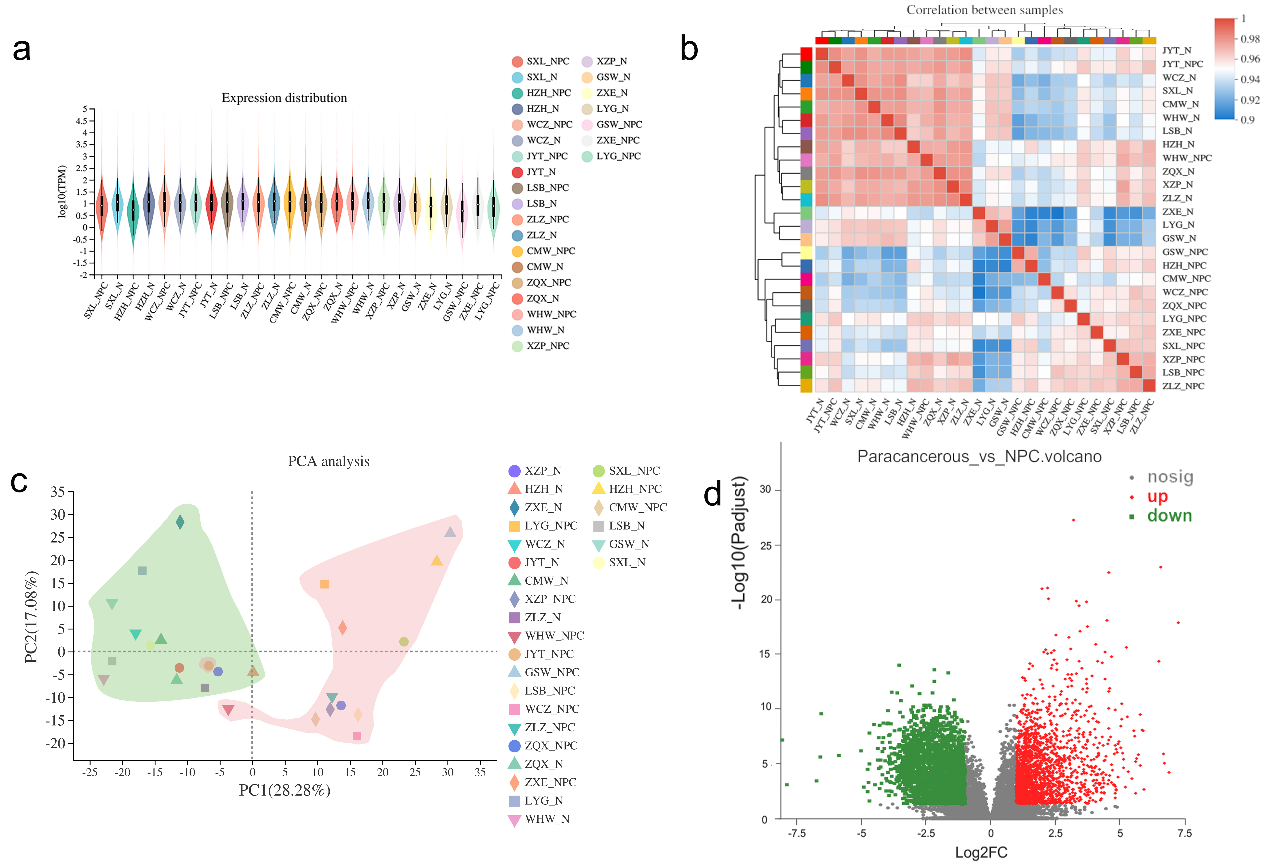


Supplementary Figure 1. The transcriptome sequencing data of NPC and paracancerous tissues. Expression distribution of all samples (a), correlation of different samples (b) and PCA analysis (c) were conducted on the sequencing data. (d) Volcano plots of detected genes in paracancerous tissues versus NPC. Red and green dots in (d) indicated significantly up- and down-regulated genes (p<0.05).


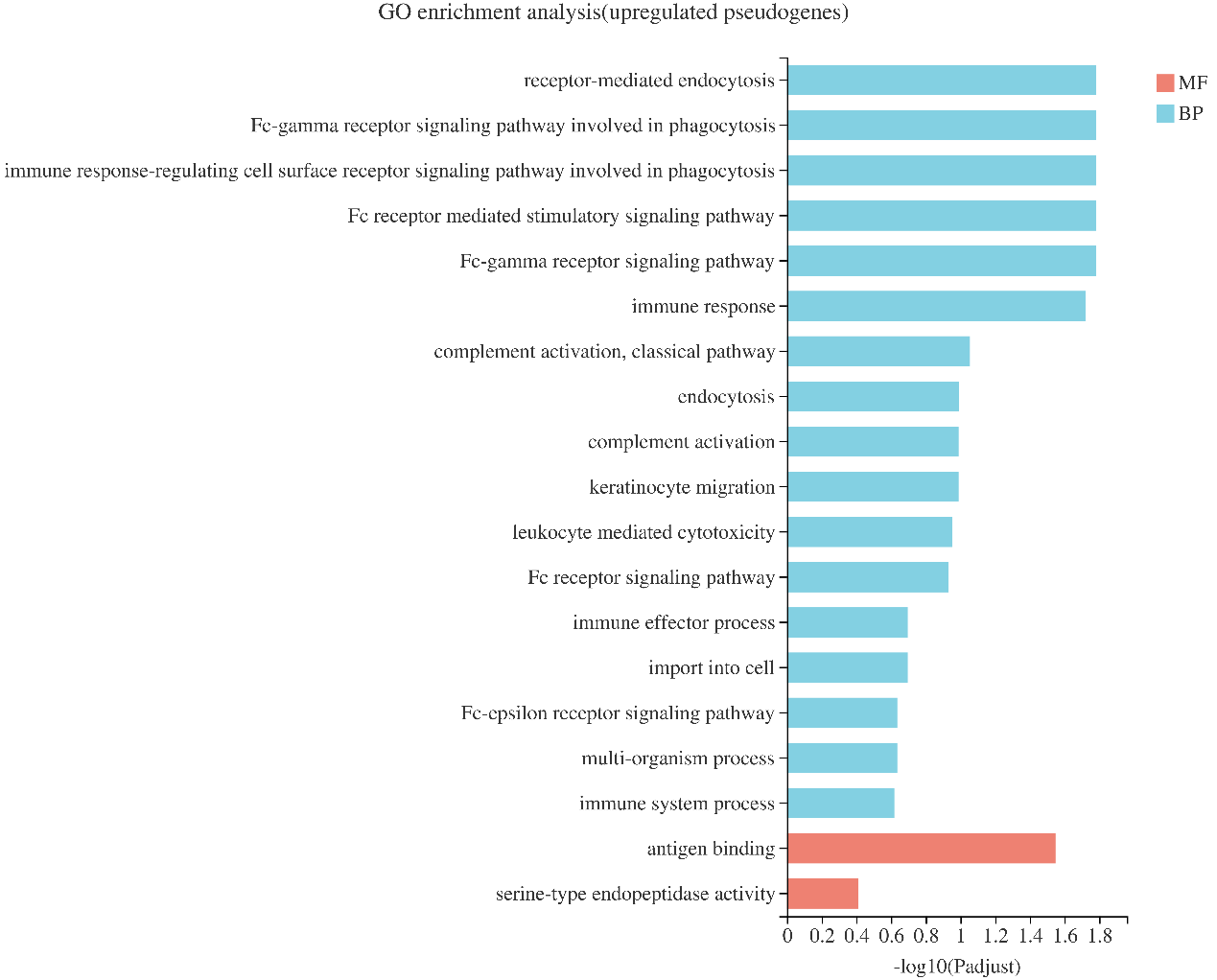


Supplementary Figure 2. The top 20 enriched GO terms of up-regulated pseudogenes belonged to BP and MF class.


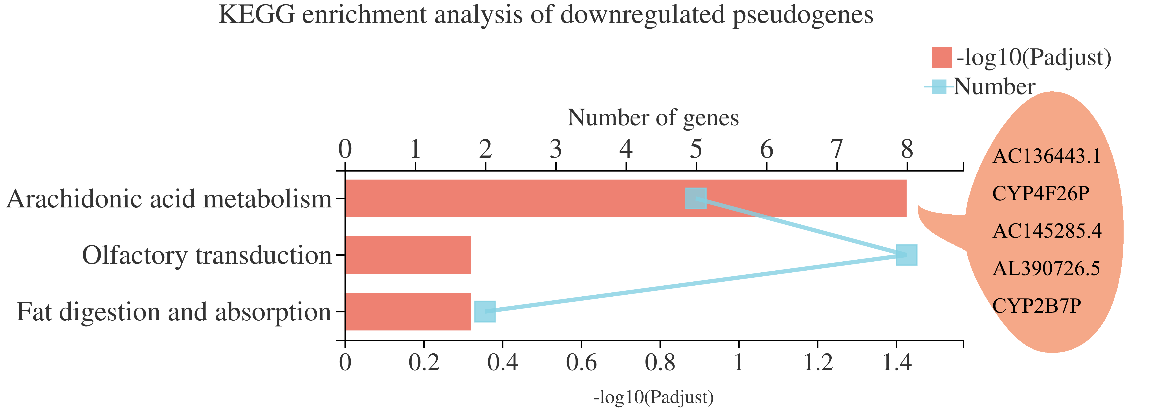


Supplementary Figure 3. KEGG pathway enrichment analysis on down-regulated pseudogenes.


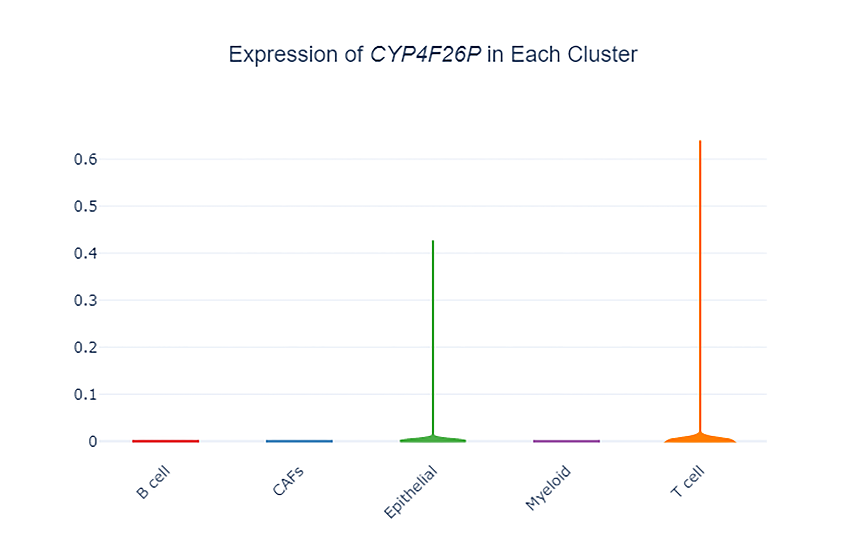


Supplementary Figure 4. *CYP4F26P* expressed in the epithelial and T cells on NPC tumors (Data source: db.cngb.org/npcatlas by Chen et al. [45]).

Supplementary Table 1. Down-regulated pseudogenes between primary NPC and paracancerous samples.

| Gene name | Log_2_FC | Padjust |
| --- | --- | --- |
| TPTEP1 | -1.475 | 0.0001 |
| FMO6P | -1.251 | 0.0246 |
| ZNF137P | -1.050 | 0.0190 |
| HMGB1P1 | -1.713 | 0.0434 |
| CYP2G1P | -1.952 | 0.0168 |
| AC211486.1 | -2.924 | 0.0000 |
| FRMPD2B | -3.053 | 0.0000 |
| SNX29P1 | -1.474 | 0.0201 |
| ALMS1P1 | -1.166 | 0.0063 |
| INTS4P1 | -1.170 | 0.0066 |
| FAM86FP | -1.249 | 0.0012 |
| CMAHP | -1.002 | 0.0001 |
| METTL15P1 | -1.342 | 0.0026 |
| GIMAP3P | -1.677 | 0.0358 |
| AC092656.1 | -1.989 | 0.0412 |
| MEIS3P1 | -1.026 | 0.0031 |
| OR7E22P | -1.781 | 0.0057 |
| OR7E14P | -1.390 | 0.0021 |
| UNC93B5 | -2.445 | 0.0485 |
| AC013268.1 | -1.311 | 0.0272 |
| DTX2P1 | -3.226 | 0.0279 |
| KRT18P59 | -1.544 | 0.0423 |
| MEIS3P2 | -1.550 | 0.0003 |
| NPIPB10P | -1.966 | 0.0074 |
| ZNF300P1 | -2.032 | 0.0071 |
| RNU1-36P | -2.960 | 0.0000 |
| CCDC162P | -1.197 | 0.0012 |
| LRRC37A5P | -2.567 | 0.0000 |
| HLA-J | -1.381 | 0.0054 |
| ZNF204P | -1.389 | 0.0013 |
| AC211476.1 | -2.069 | 0.0000 |
| SERPINB11 | -3.611 | 0.0001 |
| RNU6-696P | -3.880 | 0.0000 |
| TTLL13P | -1.883 | 0.0025 |
| AC103591.2 | -2.495 | 0.0075 |
| RRM2P3 | -1.455 | 0.0046 |
| KRT42P | -2.090 | 0.0000 |
| LRRC37A11P | -1.681 | 0.0003 |
| SEM1P1 | -2.254 | 0.0000 |
| OR7E122P | -1.162 | 0.0009 |
| SLC25A5P1 | -1.674 | 0.0370 |
| SDHAP2 | -1.294 | 0.0000 |
| HMGA1P7 | -4.330 | 0.0000 |
| AL355312.1 | -1.834 | 0.0046 |
| MTCO2P33 | -1.485 | 0.0065 |
| AL049697.3 | -2.563 | 0.0270 |
| HIST2H2BA | -2.385 | 0.0000 |
| AC104162.1 | -3.292 | 0.0000 |
| MUC20P1 | -2.492 | 0.0000 |
| AC011447.1 | -2.154 | 0.0272 |
| SNX18P3 | -1.950 | 0.0000 |
| PGM5P4 | -1.655 | 0.0002 |
| CCT8P1 | -1.076 | 0.0005 |
| ZBTB45P2 | -1.461 | 0.0318 |
| CYP4F26P | -2.545 | 0.0015 |
| RPL39P40 | -2.510 | 0.0000 |
| ABHD17AP6 | -2.009 | 0.0000 |
| GTF2IP7 | -1.365 | 0.0280 |
| ALG1L8P | -2.174 | 0.0005 |
| CES1P1 | -3.375 | 0.0000 |
| AC068533.2 | -2.889 | 0.0255 |
| AC005077.4 | -1.440 | 0.0038 |
| SLC47A1P2 | -2.428 | 0.0107 |
| AC079781.1 | -3.162 | 0.0008 |
| AC087163.2 | -1.761 | 0.0062 |
| RPL21P108 | -2.067 | 0.0038 |
| AL138787.1 | -2.747 | 0.0000 |
| SEC1P | -1.614 | 0.0018 |
| CTAGE3P | -2.663 | 0.0292 |
| EEF1A1P14 | -1.266 | 0.0450 |
| MIPEPP3 | -1.314 | 0.0016 |
| CYP2T1P | -1.990 | 0.0000 |
| AC139769.1 | -1.695 | 0.0000 |
| AL390879.1 | -1.496 | 0.0001 |
| BCAS2P2 | -2.046 | 0.0000 |
| OR7E155P | -4.321 | 0.0000 |
| TEX21P | -1.494 | 0.0002 |
| AL450326.2 | -2.269 | 0.0003 |
| AL355877.1 | -3.683 | 0.0000 |
| OR7E109P | -2.636 | 0.0205 |
| AL593856.1 | -2.544 | 0.0376 |
| AC104073.2 | -2.873 | 0.0003 |
| CYP2F2P | -1.822 | 0.0237 |
| GSTA7P | -3.709 | 0.0007 |
| EEF1A1P30 | -2.400 | 0.0425 |
| AC104852.1 | -2.254 | 0.0000 |
| GOLGA2P5 | -1.079 | 0.0000 |
| CCR12P | -1.573 | 0.0142 |
| IFNWP19 | -2.558 | 0.0060 |
| RPL23AP1 | -1.109 | 0.0261 |
| RPS4XP18 | -3.249 | 0.0066 |
| RPSAP41 | -2.962 | 0.0388 |
| AC092104.1 | -3.256 | 0.0000 |
| RNF7P1 | -2.779 | 0.0326 |
| AC093010.1 | -1.508 | 0.0339 |
| AADACP1 | -1.264 | 0.0326 |
| HIST2H2BB | -1.162 | 0.0334 |
| GM2AP1 | -2.038 | 0.0028 |
| CRYZL2P | -1.311 | 0.0009 |
| AC008897.1 | -2.154 | 0.0439 |
| RPS3AP34 | -2.267 | 0.0015 |
| OR7E29P | -2.520 | 0.0369 |
| SNRPCP10 | -2.436 | 0.0000 |
| RPL5P24 | -2.219 | 0.0253 |
| RPS4XP6 | -1.021 | 0.0240 |
| AC013356.1 | -1.065 | 0.0013 |
| RN7SL636P | -2.512 | 0.0493 |
| AC005154.2 | -1.143 | 0.0007 |
| SNRPCP3 | -2.273 | 0.0018 |
| RPS3AP15 | -1.735 | 0.0000 |
| AC105391.1 | -3.395 | 0.0022 |
| NPM1P27 | -1.168 | 0.0375 |
| ENPP7P10 | -1.638 | 0.0292 |
| AC003072.1 | -1.055 | 0.0164 |
| RPSAP70 | -1.291 | 0.0428 |
| SNRPCP16 | -3.115 | 0.0000 |
| ANKRD20A17P | -1.124 | 0.0358 |
| AC110998.1 | -2.295 | 0.0001 |
| TPTE2P1 | -1.240 | 0.0151 |
| SNRPCP17 | -2.736 | 0.0040 |
| AC136443.1 | -1.843 | 0.0000 |
| AC004923.1 | -1.507 | 0.0038 |
| SLC25A47P1 | -2.354 | 0.0449 |
| ATP5PBP5 | -1.283 | 0.0258 |
| STRA6LP | -1.967 | 0.0000 |
| AP002008.2 | -2.428 | 0.0230 |
| AC068587.2 | -1.746 | 0.0000 |
| ABCC6P2 | -2.998 | 0.0003 |
| OR5BA1P | -1.601 | 0.0060 |
| AC105233.3 | -3.250 | 0.0000 |
| AL590627.1 | -1.029 | 0.0048 |
| PABPC1P4 | -1.149 | 0.0028 |
| ABCC6P1 | -1.984 | 0.0002 |
| AC092745.2 | -1.777 | 0.0008 |
| CYP2B7P | -2.750 | 0.0001 |
| AC124947.2 | -2.756 | 0.0001 |
| OR7E47P | -3.014 | 0.0000 |
| AC084879.1 | -2.496 | 0.0000 |
| AC106782.1 | -1.419 | 0.0000 |
| AC091544.3 | -2.321 | 0.0482 |
| AL355075.5 | -1.633 | 0.0001 |
| AC097374.1 | -1.369 | 0.0139 |
| PPIAP46 | -2.612 | 0.0001 |
| AC009093.3 | -3.139 | 0.0000 |
| WFDC21P | -1.260 | 0.0022 |
| AC145285.4 | -2.488 | 0.0000 |
| AC133065.2 | -1.457 | 0.0015 |
| ABHD17AP5 | -1.090 | 0.0226 |
| AC069366.1 | -2.769 | 0.0005 |
| AGAP7P | -1.028 | 0.0035 |
| LRRC37A7P | -2.419 | 0.0000 |
| AC005336.1 | -1.634 | 0.0246 |
| CYP4F24P | -2.193 | 0.0365 |
| AC016582.3 | -2.124 | 0.0000 |
| CYP2G2P | -3.283 | 0.0010 |
| BMS1P7 | -2.739 | 0.0367 |
| H3F3AP2 | -1.232 | 0.0377 |
| LAPTM4BP2 | -2.349 | 0.0328 |
| AL078624.1 | -3.078 | 0.0006 |
| AC011477.6 | -2.840 | 0.0069 |
| AC098935.2 | -1.676 | 0.0275 |
| SNX29P2 | -1.743 | 0.0001 |
| AL390726.5 | -2.264 | 0.0436 |
| ENPP7P7 | -1.510 | 0.0033 |
| ADAMTS7P1 | -1.087 | 0.0022 |
| KRT89P | -2.322 | 0.0215 |
| AL445584.3 | -3.436 | 0.0000 |
| CNTNAP3P2 | -1.613 | 0.0000 |
| HYDIN2 | -2.110 | 0.0018 |
| AC107081.3 | -3.061 | 0.0000 |
| FP236315.2 | -1.092 | 0.0500 |
| CU634019.3 | -1.092 | 0.0500 |
| FP671120.3 | -1.092 | 0.0500 |
| AC068620.3 | -1.891 | 0.0006 |
| AC137936.2 | -1.943 | 0.0432 |
| AF228730.5 | -2.113 | 0.0008 |
| AC116562.4 | -2.410 | 0.0000 |
| AL139082.2 | -1.030 | 0.0014 |

FC meant the fold change between NPC and paracancerous tissues. Padjust was the P value adjusted by FDR correction with Benjamini/Hochberg. P value was determined by DESeq2.

Supplementary Table 2. Up-regulated pseudogenes between primary NPC and paracancerous samples.

| Gene name | Log_2_FC | Padjust |
| --- | --- | --- |
| MCTS2P | 1.404 | 0.0011 |
| TUBBP1 | 1.119 | 0.0002 |
| ZDHHC8P1 | 1.944 | 0.0061 |
| CCR5 | 1.449 | 0.0000 |
| AC007663.1 | 1.853 | 0.0020 |
| LILRP2 | 2.132 | 0.0351 |
| OR10AC1 | 2.839 | 0.0000 |
| CCNYL2 | 3.597 | 0.0000 |
| DHFRP1 | 1.774 | 0.0471 |
| RNA5SP389 | 2.245 | 0.0008 |
| DUXAP8 | 1.186 | 0.0037 |
| IGHV3-11 | 1.382 | 0.0156 |
| KRT16P3 | 2.776 | 0.0005 |
| APOC1P1 | 2.985 | 0.0003 |
| KRT16P1 | 2.248 | 0.0015 |
| RAET1K | 1.572 | 0.0002 |
| BTF3P7 | 1.743 | 0.0001 |
| FAM96AP2 | 3.301 | 0.0006 |
| IGKV1D-27 | 2.576 | 0.0082 |
| OR52K3P | 1.912 | 0.0058 |
| SFTA1P | 2.780 | 0.0068 |
| GBP1P1 | 1.395 | 0.0000 |
| MTND2P28 | 1.132 | 0.0007 |
| AL031846.1 | 1.376 | 0.0053 |
| MTND1P23 | 3.824 | 0.0000 |
| AC005515.1 | 1.432 | 0.0263 |
| AL160286.1 | 2.352 | 0.0083 |
| HNRNPA1P21 | 3.204 | 0.0000 |
| NAMPTP1 | 1.333 | 0.0063 |
| CXCR2P1 | 2.287 | 0.0000 |
| RPL4P6 | 1.176 | 0.0050 |
| CNOT6LP1 | 1.531 | 0.0283 |
| GTF2IRD1P1 | 1.399 | 0.0462 |
| AL445933.1 | 2.293 | 0.0004 |
| AC093311.1 | 2.080 | 0.0007 |
| MARK2P9 | 2.814 | 0.0011 |
| ANAPC1P1 | 1.724 | 0.0486 |
| SULT1C2P1 | 2.020 | 0.0271 |
| HLA-DPA3 | 1.729 | 0.0052 |
| AC097527.1 | 5.590 | 0.0000 |
| AC010980.1 | 3.060 | 0.0000 |
| EIF4HP2 | 1.802 | 0.0373 |
| ABCA17P | 1.035 | 0.0015 |
| TBC1D3P1 | 3.793 | 0.0457 |
| AC116353.1 | 3.578 | 0.0016 |
| FABP5P3 | 1.274 | 0.0427 |
| IGKV1-39 | 1.445 | 0.0057 |
| RPLP0P2 | 2.146 | 0.0000 |
| AL589743.1 | 1.077 | 0.0015 |
| ENO1P1 | 2.674 | 0.0005 |
| AC111193.1 | 2.660 | 0.0013 |
| AACSP1 | 3.394 | 0.0000 |
| OR7E28P | 4.341 | 0.0000 |
| AL606534.4 | 1.901 | 0.0370 |
| IGKV2OR22-4 | 3.286 | 0.0000 |
| IGLV1-41 | 1.066 | 0.0202 |
| AP001994.1 | 1.429 | 0.0416 |
| SIGLEC12 | 2.373 | 0.0001 |
| AC084357.3 | 3.330 | 0.0002 |
| TOMM20P2 | 2.110 | 0.0044 |
| AC112777.1 | 1.438 | 0.0236 |
| OTOAP1 | 1.235 | 0.0138 |
| PPIAP45 | 2.480 | 0.0002 |
| ZNF519P3 | 5.796 | 0.0000 |
| IGHV1OR15-2 | 2.331 | 0.0040 |
| FCGR1CP | 3.705 | 0.0000 |
| AC138207.6 | 1.520 | 0.0294 |
| ZNF887P | 2.920 | 0.0000 |
| LRRC37A9P | 2.006 | 0.0041 |
| ACTG1P22 | 2.611 | 0.0043 |
| IGHV3-69-1 | 1.525 | 0.0073 |
| AC116353.5 | 1.335 | 0.0479 |
| AL031274.1 | 1.296 | 0.0072 |

Supplementary Table 3. The 10 most significantly down-regulated pseudogenes between primary NPC and paracancerous samples.

| Gene name | Gene description | Log_2_FC | Padjust | Paracancer | NPC |
| --- | --- | --- | --- | --- | --- |
| STRA6LP | STRA6 like, pseudogene | -1.967 | 3.79934E-10 | 3.995 | 0.950 |
| AC084879.1 | novel pseudogene | -2.496 | 2.53237E-09 | 0.834 | 0.145 |
| AC116562.4 | RNA binding protein, fox-1 homolog pseudogene | -2.410 | 1.14475E-08 | 5.779 | 0.955 |
| RNU6-696P | RNA, U6 small nuclear 696, pseudogene | -3.880 | 2.2742E-08 | 1.235 | 0.082 |
| OR7E155P | olfactory receptor family 7 subfamily E member 155 pseudogene | -4.321 | 4.61234E-08 | 2.416 | 0.131 |
| RPS3AP15 | ribosomal protein S3a pseudogene 15 | -1.735 | 4.85932E-08 | 4.174 | 0.802 |
| AC104852.1 | polo-like kinase 1 pseudogene | -2.254 | 5.06282E-08 | 1.045 | 0.215 |
| FRMPD2B | FERM and PDZ domain containing 2B, pseudogene | -3.053 | 7.30332E-08 | 2.535 | 0.326 |
| AC139769.1 | zinc finger protein pseudogene | -1.695 | 1.40751E-07 | 3.198 | 0.922 |
| AC105233.3 | ribosomal protein S24 pseudogene | -3.250 | 2.3321E-07 | 5.005 | 0.553 |

Supplementary Table 4. The 10 most significantly up-regulated pseudogenes between primary NPC and paracancerous samples.

| Gene name | Gene description | Log_2_FC | Padjust | Paracancer | NPC |
| --- | --- | --- | --- | --- | --- |
| HNRNPA1P21 | heterogeneous nuclear ribonucleoprotein A1 pseudogene 21 | 3.204 | 5.96478E-28 | 0.714 | 5.276 |
| AACSP1 | acetoacetyl-CoA synthetase pseudogene 1 | 3.394 | 1.59171E-10 | 0.102 | 0.952 |
| FCGR1CP | Fc fragment of IgG receptor Ic, pseudogene | 3.705 | 1.59171E-10 | 0.147 | 1.395 |
| CCR5 | C-C motif chemokine receptor 5 (gene/pseudogene) | 1.449 | 1.02212E-08 | 10.655 | 24.517 |
| CXCR2P1 | C-X-C motif chemokine receptor 2 pseudogene 1 | 2.287 | 2.06687E-08 | 2.473 | 9.875 |
| AC097527.1 | pseudogene similar to part of anaphase promoting complex subunit 1 | 5.590 | 3.18512E-08 | 0.007 | 0.298 |
| OR7E28P | olfactory receptor family 7 subfamily E member 28 pseudogene | 4.341 | 3.93842E-08 | 0.060 | 1.190 |
| CCNYL2 | cyclin Y-like 2 (pseudogene) | 3.597 | 4.0839E-08 | 0.026 | 0.199 |
| IGKV2OR22-4 | immunoglobulin kappa variable 2/OR22-4 (pseudogene) | 3.286 | 3.79416E-07 | 6.414 | 41.237 |
| GBP1P1 | guanylate binding protein 1 pseudogene 1 | 1.395 | 1.29516E-06 | 2.845 | 7.584 |
